# Factors associated with viral load suppression in pregnant and postpartum women living with HIV in Rwanda: an open-observational cohort study

**DOI:** 10.3389/fpubh.2025.1544165

**Published:** 2025-04-09

**Authors:** Athanase Munyaneza, Hae-Young Kim, Qiuhu Shi, Ellen Brazier, Jonathan Ross, Benjamin Muhoza, Faustin Kanyabwisha, Gallican Kubwimana, Gad Murenzi, Denis Nash, Kathryn Anastos, Marcel Yotebieng

**Affiliations:** ^1^Research for Development (RD Rwanda), Kigali, Rwanda; ^2^Institutional Centers for Clinical and Translational Research, Boston Children's Hospital, Boston, MA, United States; ^3^Department of Public Health, New York Medical College, Valhalla, NY, United States; ^4^Institute for Implementation Science in Population Health, City University of New York, Graduate School of Public Health and Health Policy, New York, NY, United States; ^5^Division of General Internal Medicine, Albert Einstein College of Medicine/Montefiore Medical Center, Bronx, NY, United States

**Keywords:** pregnant and postpartum women, HIV, viral load suppression, IeDEA, Kigali, Rwanda

## Abstract

**Introduction:**

Achieving and maintaining HIV viral load suppression (VLS) in pregnant and postpartum women living with HIV (WLWH) is critical for their health and to prevent mother-to-child transmission. However, data on VLS in this population are limited. This study aimed to evaluate the prevalence and factors associated with VLS among pregnant and postpartum WLWH in Rwanda within 12 months of enrolling in antenatal care.

**Methods:**

An open observational cohort study was conducted using routine clinical data from 10 Rwandan HIV clinics in Kigali City. Data from WLWH on ART who became pregnant and were referred to PMTCT services between 2012 and 2020 were analyzed. The primary outcomes were the proportion of WLWH achieving VLS (viral load (VL) <1,000 or <200 copies/mL) within 12 months of antenatal registration. Logistic regression models assessed associations of VLS with socio-demographic and clinical characteristics.

**Results:**

Among 1,002 WLWH, 532 (53%) had documented VL results. The mean age was 30.4 years, with 60% aged 25–34 years. Most (83.7%) were primigravida, and 67% initiated ART before pregnancy. At antenatal care enrollment, 58% had a CD4 count ≥500 cells/uL. Within 12 months, 92% had VL <1,000 copies/mL and 87% had a VL <200 copies/mL. Advanced HIV disease (WHO stage 3 and 4) and lower CD4 counts were associated with lower odds of VLS.

**Conclusion:**

Among those with a recorded VL results, nine out of 10 had a VLS, particularly those without advanced HIV disease. The findings underscore the need for targeted interventions for WLWH with advanced HIV entering antenatal care.

## Introduction

Achieving and maintaining viral load suppression (VLS) in pregnant and postpartum women living with HIV (WLWH) remains a global public health priority (1). The Joint United Nations Programme on HIV/AIDS (UNAIDS) VLS target for ending the epidemic is for 95% of pregnant and postpartum WLWH on antiretroviral treatment (ART) to achieve and sustain VLS both before delivery throughout and beyond the postpartum period (2, 3). While there has been substantial progress in the scale up of ART among pregnant and postpartum WLWH through the prevention of mother-to-child transmission (PMTCT) programs around the globe (4), studies indicate persistent challenges in achieving and sustaining VLS among this population (5, 6). Without adequate viral suppression (VS), pregnant women face higher risks of HIV-related morbidity, mortality, and increased HIV transmission to the infant, which can result in early infant mortality and long-term health issues due to mother-to-child transmission (MTCT) (7). Although high rates of VS are observed during pregnancy, the postpartum period presents greater challenges due to factors like healthcare access, adherence, and breastfeeding, particularly in Southern Africa. This period increases the risk of HIV transmission to the infant and poses additional health risks to the mother (8). Existing VLS data are primarily focused on WLWH in general, with only 76% achieving VLS by 2022 among those on ART (9).

In sub-Saharan Africa (SSA), where the HIV burden is highest, there is little data on VLS among pregnant and postpartum WLWH because the scale up of routine viral load monitoring has been slow, and pregnant and postpartum WLWH are not sufficiently recognized as a priority population (5). Data on VLS among this population primarily comes from research studies rather than routine care programs, and estimates vary widely, depending on the thresholds used for VLS (5, 10–13).

Available data suggest that even with conservative cut-offs (i.e., < 1,000 copies/mL), VLS among this population is far below UNAIDS targets. A cross sectional analysis of Population-Based HIV Impact Assessment surveys across 10 SSA countries using data collected between 2015 and 2018 found that, of 1,685 pregnant or breastfeeding WLHIV regardless of their ART status, only 63.8% WLWH had HIV viral load < 1,000 copies/mL at the study visit (6). Considerable variation by region, ranging from 40.1% in Central and West Africa to 66.2% in East Africa and 76.2% in Southern Africa (6). Similarly, a population-representative cross sectional study conducted in South Africa (SA) found that, Of 10 052 of pregnant WLWH attending antenatal care on ART, only 79.5% were virally suppressed using a threshold of viral load ≤ 1,000 copies/mL, with only 56.2% being virally suppressed using a lower threshold of 50 copies/mL (10).

As of 2022, Rwanda was one of only five countries meeting UNAIDS targets for VLS among all people living with HIV on ART (4). However, disparities persist among subpopulations. Few data are available on VLS among pregnant and postpartum Rwandan WLWH as neither UNAIDS nor national HIV program data disaggregate VLS data by pregnancy or postpartum status. To our knowledge, the only estimate of VLS among this population are from a 2013 to 2014 observational prospective cohort study that assessed 24-months HIV-free survival among infants born to 608 WLWH enrolled in PMTCT programs (14). This study found that 85% had VLS using a threshold of < 1,000 copies/mL, but only half had an undetectable viral load ( ≤ 20 copies/mL) at enrollment (14). In the current study, we aimed to estimate the prevalence of VLS at 12-months after the initial documentation of a pregnancy among WLWH in Rwanda and to explore factors associated with VLS among this population.

## Methods

### Study design and data source

We conducted an open observational cohort study using routine clinical data sourced from HIV care clinics in Rwanda that participate in the Central Africa International epidemiology Databases to Evaluate AIDS (CA-IeDEA) (15). In Rwanda, CA-IeDEA collects routine care data from 12 HIV care clinics, including ten that provide PMTCT services. Of these, eight are urban clinics located in Kigali City and two are in rural areas in the Eastern and Northern Provinces of Rwanda. The data for this study were extracted from the PMTCT module of OpenMRS, an electronic medical record system used in all HIV care clinics in Rwanda. The Rwanda Ministry of Health introduced the PMTCT module in early 2018, and data were entered retrospectively for WLWH entering PMTCT care. Non-HIV care data including socio-demographic and obstetric information was manually extracted from antenatal, delivery, and postpartum care registries for women who tested HIV-positive.

### Study population

We considered data from WLWH on ART who registered for PMTCT services (including antenatal care, delivery, or postpartum care) at IeDEA sites between April 2012, when Rwanda launched Option B+, a World Health Organization (WHO) guidelines for pregnant WLHIV, and June 2020. A period that extends both before and after the implementation of universal test and teat (UTT) policy in the country. To be included in the analysis, WLWH had to have a recorded viral load results between the initial documentation of pregnancy and 12-months after.

### Study variables

The main outcome of interest was VLS within 12 months of the antenatal care registration at the site (12-months VLS). For individuals with multiple recorded viral load results during the evaluation period, the most recent one was used to assess suppression. We used two cut-offs for defining VLS, including VL < 1,000 copies/mL in accordance with UNAIDS standards (3) and VL < 200 copies/mL in accordance with Rwanda's 2018 National Guidelines for the Prevention and Management of HIV and STIs which also recommends that pregnant WLHIV have a VL test 3 months after ART initiation, then every 6 months thereafter (16). Independent variables included: (1) socio-demographic information at the time of entry into PMTCT, including age (categorized as 15–24 years, 25–34 years, and ≥35 years), marital status (married vs. not married), body mass index (BMI) in kg/m^2^ (categorized as underweight: < 18.5, normal: 18.5–24.9, overweight: 25–29.9, and obese ≥30); (2) HIV clinical information, including HIV disclosure status, WHO stage (categorized as stages 1 or 2 vs. stages 3 or 4), timing of ART initiation (before vs. after pregnancy), and CD4 count (categorized as < 200, 200–500 and ≥500 cells/μl); and (3) Obstetric and antenatal information, including gravida and gestational week of pregnancy at PMTCT registration. We used the date of ART initiation and date of the last menstrual period (LMP) to determine the timing of ART start as before vs. after pregnancy, and we determined gestational age based on LMP date and the date of PMTCT registration. Before and after the implementation of (UTT) policy in the country was defined as pregnancy registration before date or after.

### Data analysis

We summarized individual characteristics using means, standard deviations (SD) or medians and interquartile ranges (IQR) for continuous variables and count and percentages for categorical variables. Logistic regression models were used to examine associations between characteristics of WLWH at PMTCT registration and VLS by 12-months after registration. Variables found to be statistically associated (*p* < 0.05) with VLS in bivariate analyses were included in a multivariable logistic regression model to estimate adjusted Odds ratios (aOR) and 95% confidence intervals (95%CI). All analyses were conducted using IBM SPSS statistical software, version 21.

### Ethical approval

The CA-IeDEA study was reviewed and approved by both the Rwanda National Ethics Committee (RNEC) with reference number 355/RNEC/2018 and the Albert Einstein College of Medicine Institutional Review Board. A waiver for informed consent was granted for the analysis of de-identified, routinely collected data.

## Results

### Data flow

**Figure 1:**
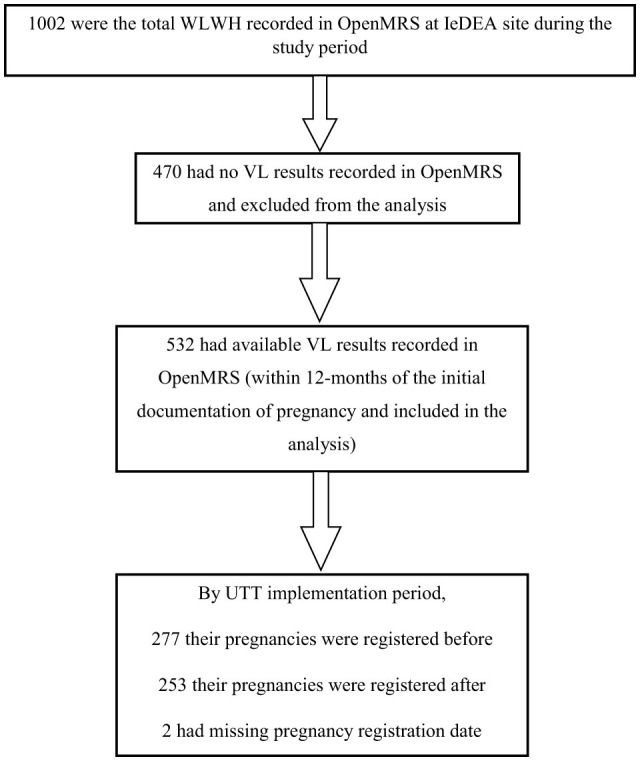
Data flow chart.

Data were available in OpenMRS for 1,002 WLWH enrolling in PMTCT from 2012 to 2020 at the 10 CA-IeDEA sites included in the study. Of those, 470 (47%) had no recorded VL results between their registration and 12 months later and were excluded from further analyses. Among those with available VL results, 277 (52.1%) had their pregnancy registration completed before the implementation of UTT, while 253 (47.6%) completed it after the UTT implementation and 2 (0.4%) had a missing registration date (Figure 1). The demographic characteristics of those with and without recorded VL result were similar.

### Characteristics of study population

Among 532 pregnant WLWH with available VL results, mean age at the time of PMTCT registration was 30.4 years (SD: 5.5), with 60% (*n* = 319) aged 25–34 years and 21% (*n* = 112) aged 24 years or younger (Table 1). The majority of WLWH (71%, *n* = 228) were married, and most (58%, *n* = 270) had a normal BMI (18.5–24.9 kg/m^2^). The majority of WLWH (84%, *n* = 436) were experiencing their first pregnancy, and most (62%, *n* = 296) had enrolled in PMTCT between the 14th and 28th weeks of pregnancy. Two-thirds (67%, *n* = 356) had initiated treatment prior to their current pregnancy. Of those with HIV disease staging recorded (*n* = 520), 90% (*n* = 468) had early-stage disease (WHO staging 1 or 2). Of those with a CD4 cell count recorded (*n* = 526), the median CD4 count was 542, IQR: (374–722) cells/μl and more than half (58%, n=303), had a CD4 count of 500 cells/μl or higher.

**Table 1:** Characteristics of WLWH enrolling in PMTCT at 10 health centers at Rwanda IeDEA sites between 2012 and 2020.

**Characteristics**	**n**	**%**
**ART start time (*****N*** = **530)**		
Before pregnant	356	67.2
During or after pregnant	174	32.8
**Age (*****N*** = **532)**		
15–24 years	84	15.8
25–34 years	319	60
35–44 years	128	24.1
> 44 years	1	0.2
**Marital status (*****N*** = **477)**		
Not married	139	29.1
Married	338	70.9
**BMI (*****N*** = **463)**		
18.5 kg/m^2^	27	5.8
18.5–24.9 kg/m^2^	270	58.3
25–29.9 kg/m^2^	111	24
≥30 kg/m^2^	55	11.9
**Gravida (*****N*** = **521)**		
Primigravida	436	83.7
Multigravida	85	16.3
**HIV disclosure (*****N*** = **231)**		
Disclosed	184	79.7
Not disclosed	47	20.3
**HIV disease stage (*****N*** = **520)**		
WHO stage 1 or 2	468	90
WHO stage 3 or 4	52	10
**CD4 count (*****N*** = **526)**		
< 200 cells/μl	27	5.1
200–499 cells/μl	196	37.3
≥500 cells/μl	303	57.6
**Gestational age at PMTCT registration (*****N*** = **476)**		
< 14 weeks	79	16.6
14–28 weeks	296	62.2
>28 weeks	101	21.2
**Pregnancy registration before or after the UTT period**		
**(*****N*** = **532)**		
Pregnancy registration before UTT date	277	52.3
Pregnancy registration after UTT date	253	47.7
**Viral load values measured within the first 12-months**		
**(cutoff of**<**1,000 copies/mL;** ***N*** = **532)**		
< 1,000 copies/mL	489	91.9
≥1,000 copies/mL	43	8.1
< 200 copies/mL	464	87.2
>200 copies/mL	68	12.8

N, count; %, Percentage.

### Prevalence of VLS among pregnant and postpartum WLWH at Rwanda IeDEA sites

Within 12 months of entering PMTCT program, 92% (*n* = 489) of WLWH with a viral load had a VL < 1,000 copies/mL, and 87% (*n* = 464) had VL < 200 copies/mL (Table 1).

### Factors associated with VLS among pregnant and postpartum WLWH at the Rwanda IeDEA sites

In bivariate analyses, using a suppression threshold of < 1,000 copies/ml, we did not observe any statistically significant differences in 12-months VLS between those who initiated ART after pregnancy compared with those who initiated ART before (OR 1.04, 95% CI: 052, 1.97; *p* = 0.96).

Participants with WHO stage 3 or 4 were less likely to be suppressed by 12 months compared with those with WHO stage 1 or 2 (OR 0.37, 95% CI: 0.16, 0.83; *p* = 0.02; Table 2). Similarly, compared to those with a CD4 count ≥500 cells/μl, participants with a CD4 count < 200 cells/μl and those with a CD4 count of 200–499 cells/μl also had lower odds of 12 months VL < 1,000 copies/mL (OR 0.22, 95% CI: 0.07, 0.68; *p* = 0.01 and OR 0.41, 95% CI: 0.20, 0.81; *p* = 0.01, respectively).

**Table 2:** Factors associated with VLS (cutoff of <1,000 copies/mL) among pregnant and postpartum WLWH at Rwanda IeDEA site.

	**Bivariate analysis**				**Multivariable analysis**	
**Characteristics**	<**1,000 copies/mL (*****n*****, %)**	≥**1,000 copies/mL (*****n*****, %)**	**OR, 95% CI**	***p*** **-value**	**aOR (95% CI)**	***p*** **-value**
**ART start time**						
Before pregnant	327 (91.9)	29 (8.1)	Ref		
During or after pregnant	160 (92)	14 (8)	1.014 (0.52, 1.97)	0.96		
**Age**						
15–24 years	77 (91.7)	7 (8.3)	0.97 (0.40, 2.33)	0.95		
25–34 years	293 (91.8)	26 (8.2)	Ref		
≥35 years	119 (92.2)	10 (7.8)	1.05 (0.49, 2.25)	0.88		
**Marital status**						
Not married	126 (90)	13 (9.4)	0.84 (0.42, 1.68)	0.62		
Married	311 (92)	27 (8)	Ref		
**BMI**						
< 18.5 kg/m^2^	25 (92.6)	2 (7.4)	1 (0.22, 4.52)	1		
18.5–24.9 kg/m^2^	250 (92.6)	20 (7.4)	Ref		
25–29.9 kg/m^2^	101 (91)	10 (6)	0.80 (0.36, 1.78)	0.59		
≥30 kg/m^2^	49 (89.1)	6 (10.9)	0.65 (0.25, 1.71)	0.38		
**Gravida**						
Primigravida	401 (92)	35 (8)	Ref		
Multigravida	79 (92.9)	6 (7.1)	1.14 (0.46, 2.82)	0.76		
**HIV disease stage**						
WHO stage 1 or 2	434 (92.7)	34 (7.3)	Ref		Ref	
WHO stage 3 or 4	43 (82.7)	9 (17.3)	**0.37 (0.16, 0.83)**	**0.02**	**0.43 (0.19, 0.98)**	**0.045**
**CD4 count**						
< 200 cells/μl	22 (81.5)	5 (18.5)	**0.22 (0.07, 0.68)**	**0.01**	**0.26 (0.08, 0.82)**	**0.021**
200–499 cells/μl	174 (88.8)	22 (11.2)	**0.41 (0.20, 0.81)**	**0.01**	**0.42 (0.21, 0.84)**	**0.015**
≥500 cells/μl	288 (95)	15 (5)	Ref		**Ref**	
**Gestational age at PMTCT registration**						
< 14 weeks	74 (93.7)	5 (6.3)	1.36 (0.50, 3.68)	0.53		
14–28 weeks	271 (91.6)	25 (8.4)	Ref		
>28 weeks	94 (93.1)	7 (6.9)	1.23 (0.51, 2.95)	0.63		

Ref, reference category; bold variables, Variables with statistical significance.

Compared with those with WHO stage 1 and 2, those with stage 3 and 4 had lower odds of VLS within 12-months (aOR 0.43, 95% CI: 0.19, 0.98). Compared with those with a CD4 count ≥500 cells/μl, those with a CD4 count ≤ 200 cells/μl and a CD4 count of 200–499 cells/μl also had reduced odds of 12-months VL < 1,000 copies/mL (aOR: 0.26, 95% CI: 0.08, 0.82 and aOR 0.42, 95% CI: 0.21, 0.98, respectively). Results were not sensitive to the threshold used for VLS, as unadjusted and adjusted ORs were nearly identical when using the lower VLS threshold of ≤ 200 copies/mL (Table 3).

**Table 3:** Factors associated with VLS (cutoff of <200 copies/mL) among pregnant and postpartum WLWH at Rwanda IeDEA site.

	**Bivariate analysis**				**Multivariable analysis**	
**Characteristics**	<**200 copies/mL (*****n*****, %)**	>**200 copies/mL (*****n*****, %)**	**OR, 95% CI**	***p*** **-value**	**aOR (95% CI)**	***p*** **-value**
**ART start time**						
Before pregnant	309 (86.8)	47 (13.2)				
During or after pregnant	153 (87.9)	21 (12.1)	1.01 (0.51, 1.97)	0.96		
**Age**						
15–24 years	281 (88.1)	38 (11.9)	Ref			
25–34 years	71 (84.5)	13 (15.5)	0.97 (0.40, 2.33)	0.95		
≥35 years	112 (86.8)	17 (13.2)	1.05 (0.49, 2.25)	0.88		
**Marital status**						
Not married	120 (86.3)	19 (13.7)	0.84 (0.42, 1.63)	0.62		
Married	297 (87.9)	41 (12.1)	Ref			
**BMI**						
< 18.5 kg/m^2^	23 (85.2)	4 (14.8)	1 (0.22, 4.52)	0.83		
18.5–24.9 kg/m^2^	243 (90)	27 (10)	Ref			
25–29.9 kg/m^2^	95 (85.6)	16 (14.4)	0.80 (0.37, 1.79)	0.59		
≥30 kg/m^2^	46 (83.6)	56 (12.1)	0.65 (0.25, 1.71)	0.38		
**Gravida**						
Primigravida	380 (87.2)	56 (12.8)	Ref			
Multigravida	76 (89.4)	9 (10.6)	1.15 (0.47, 2.82)	0.77		
**HIV disease stage**						
WHO stage 1 or 2	415 (88.7)	53 (11.3)	Ref			
WHO stage 3 or 4	37 (71.2)	15 (28.8)	**0.37 (0.17, 0.83)**	**0.016**	**0.43 (0.2, 0.98)**	**0.045**
**CD4 count**						
< 200 cells/μl	19 (70.4)	8 (29.6)	**0.23 (0.07, 0.69)**	**0.009**	**0.27 (0.09, 0.82)**	**0.021**
200–499 cells/μl	159 (81.1)	37 (18.9)	**0.41 (0.21, 0.81)**	**0.011**	**0.43 (0.21, 0.85)**	**0.015**
≥500 cells/μl	281 (92.7)	22 (7.3)	Ref			
**Gestational age at PMTCT registration**						
< 14 weeks	73 (92.4)	6 (7.6)	1.36 (0.51, 3.69)	0.539		
14–28 weeks	260 (87.8)	36 (12.2)	Ref			
>28 weeks	85 (84.2)	16 (15.8)	1.24 (0.52, 2.96)	0.63		

Ref, reference category; Bold variables, Variables with statistical significance.

## Discussion

Addressing the scarcity of data on VLS among pregnant and postpartum Rwandan WLWH, this study estimated the prevalence of VLS within 12-months of PMTCT entry at 10 HIV clinics in Rwanda participating in the CA-IeDEA, along with factors associated with VLS. We found that viral load is not routinely recorded in OpenMRS among this key group with just over half of women with at least one viral load result between PMTCT registration and 12-months after. Among those with at least one viral load results during the evaluation period, 92% had a VL < 1,000 copies/mL, and 87% < 200 copies/mL.

While data on VLS among pregnant and postpartum WLWH in Rwanda are limited, our findings align with a prior study in Rwanda, which found that 85% of pregnant and early postpartum WLWH had a VLS (< 1,000 copies/mL) (14). Our findings are also consistent with data on VLS in the general population of Rwanda, including national data from the Rwanda Ministry of Health and other studies reporting virological suppression rates of 90% to 91% among all patients on ART (17, 18) and 93% among the general population of WLWH and 78% among WLWH of reproductive age (19). Although the VLS prevalence observed in our study is slightly below UNAIDS's target of 95% (2, 3), it is considerably higher than those from studies in SSA among pregnant and postpartum WLWH in general, that reported a VLS of 64% (6). Additionally, our findings exhibit slight variations when compared to a study conducted in the Democratic Republic of Congo (DRC), which reported a VLS prevalence of 67% (< 1,000 copies/mL) among pregnant and postpartum WLWH on ART up to 12 months post-delivery (11) as well as a South African study indicating a VLS of 56% (< 50 copies/mL) (10).

These disparities in VLS rates could be attributed to: (1) differences in study designs (our study used a 12-months period prevalence while those other studies evaluated point prevalence), (2) differences in ART delivery performance (Rwanda is one of the five countries to have reached the UNAIDS targets of 95/95/95 in general population), routine monitoring, adherence support, and integration of HIV care with maternal health services, which may contribute to the high VLS rates observed, (3) to selection bias (only 53% of eligible WLWH had a recorded viral load measure). While our results indicate that VLS among this population is high, which is promising for efforts to reduce vertical transmission of HIV in Rwanda, understanding these distinctions is crucial for contextualizing and interpreting VLS outcomes.

Interestingly, we found no difference in 12-months VLS between WLWH who initiated treatment before vs. after pregnancy (*p* = 0.96). This aligns with findings from studies in Kenya and Zimbabwe where VLS did not differ between WLWH initiating ART before pregnancy and those initiating ART during pregnancy (20, 21). This contrasts with findings from SA (10, 22) and the DRC (11), highlighting the complex relationship between treatment duration and VLS. It emphasizes the critical role of additional support beyond the time to ART initiation in achieving VLS among pregnant and postpartum WLWH.

In our study, the only strong factor associated with VLS was advanced HIV disease at PMTCT registration, whether measured by WHO stage or CD4 count. While advanced disease is linked to lower VLS, it is likely a consequence of delayed HIV diagnosis and treatment rather than a direct predictor. This aligns with similar findings from SA (10), Ethiopia (23) and Tanzania (24), reinforcing important of early diagnosis of HIV particularly among women of reproductive age. Healthcare providers should pay particular attention to individuals with advanced HIV disease, and as recommended by WHO (25), tailored interventions and close monitoring should be implemented for optimal ART outcomes.

Several limitations of our study are worth noting. Our data came solely from primary health facilities most of which were located in Kigali, and none of the sites in our study were private clinics, district hospitals or referral-level facilities. While primary-level health centers are the primary providers of routine HIV care in Rwanda, our findings may not be generalizable to WLWH who are in care at other types of clinics. Additionally, most clinics in our study were located in Kigali City, and it is possible that the services and support provided to WLWH during pregnancy and the postpartum period including ART delivery, routine VL monitoring, community-based adherence support, and integrated PMTCT services in Kigali is not representative of care throughout the country. Additionally, while previous studies have identified associations between younger maternal age, higher gravidity, and non-suppression, our study did not observe these relationships. Several factors could explain these discrepancies, including differences in healthcare access, adherence support, or the characteristics of our sample. These limitations suggest the need for further research to explore these findings in greater depth and clarify the potential contributing factors. In additional, a key limitation of our study is the missing VL data for almost half of the participants, which may have affected the VLS analysis. Additionally, the lack of data on variables like adherence, maternal education, and socio-economic status further limits the findings. Lastly, our study data were collected before dolutegravir became the standard of care in Rwanda, so its impact on viral suppression was not assessed. Future research should include dolutegravir to determine its potential effects on VLS among pregnant and postpartum women living with HIV.

These limitations notwithstanding, our study also had several important strengths. Utilizing real-world service delivery data. Our study provides important insights into the prevalence of VLS among pregnant and postpartum WLWH in Rwanda, as well as factors associated with VLS in this population. We utilized data from 10 HIV clinics, over an 8-year period when Option B+ was in effect in Rwanda, encompassing both the pre and post implementation of the UTT. Lastly, the consistency of our results with other studies in various countries and populations, which focus on advanced clinical stages of HIV and lower CD4 counts as predictors of VLS among pregnant and postpartum WLWH, strengthens the reliability and generalizability of these findings.

## Conclusion

This study provides valuable information on the prevalence of VLS among pregnant and postpartum WLWH at Rwanda IeDEA sites. It highlights a substantial proportion of pregnant and postpartum WLWH achieving VLS in their first year in the PMTCT care. It also highlights the sub-optimal viral load suppression among those presenting with advanced HIV disease, a group that should be prioritized for support if the UNAIDS targets of 95% VLS among pregnant and postpartum WLWH on ART is to be reached.

## Data Availability

The raw data supporting the conclusions of this article will be made available by the authors, without undue reservation.
